# Clinical Characteristics of Japanese Cases of Anti-Metabotropic Glutamate Receptor Type 1 Antibody-Associated Cerebellar Ataxia

**DOI:** 10.1007/s12311-026-02094-z

**Published:** 2026-09-30

**Authors:** Nobuaki Yoshikura, Akio Kimura, Akira Takekoshi, Keizo Yasui, Shoko Yamashita, Keisuke Yoshikawa, Kento Sakashita, Reiji Koide, Yuki Kobayashi, Kazuki Yamada, Hiroaki Yaguchi, Ichiro Yabe, Takahiro Maku, Haruko Nakamura, Shutaro Shibata, Haruka Takami, Hideki Mori, Takayoshi Shimohata

**Affiliations:** 1 Department of Neurology, Gifu University Graduate School of Medicine, 1-1 Yanagido, Gifu, 501-1194 Japan; 2 Department of Neurology, Japanese Red Cross Aichi Medical Center, Nagoya Daini Hospital, Nagoya, Japan; 3 Department of Neurology, Kindai University Faculty of Medicine, Sakai, Osaka Japan; 4 Department of Neurology, National Hospital Organization Hyogo-Chuo National Hospital, Sanda, Japan; 5 Division of Neurology, Department of Medicine, School of Medicine, Jichi Medical University, Shimotsuke, Japan; 6 Department of Neurology and Neurological Science, Institute of Science, Tokyo, Japan; 7 Department of Neurology, Hakodate Municipal Hospital, Hakodate, Japan; 8 Department of Neurology, Faculty of Medicine, Graduate School of Medicine, Hokkaido University, Sapporo, Japan; 9 Department of Neurology, Fuji City General Hospital, Fuji City, Japan; 10 Department of Neurology and Stroke Medicine, Graduate School of Medicine, Yokohama City University, Yokohama, Japan; 11 Department of Neurology, Kouseiren Takaoka Hospital, Takaoka, Japan; 12 Department of Pediatrics, The Jikei University School of Medicine, Tokyo, Japan; 13 Department of Neurology, Niigata Prefectural Shibata Hospital, Shibata, Japan

**Keywords:** MGluR1, Autoimmune cerebellar ataxia, COVID-19, Asymmetrical ataxia, Japanese cohort

## Abstract

Anti-metabotropic glutamate receptor type 1 (mGluR1) antibody-associated cerebellar ataxia is a rare autoimmune cerebellar ataxia. Although its clinical spectrum has been recognized mainly from Western cohorts, its features in other populations remain uncharacterized. We investigated the clinical characteristics of this disease in a Japanese cohort with reference to overseas cases. We retrospectively reviewed patients testing positive for anti-mGluR1 antibodies in our laboratory between 2018 and 2025, comparing their clinical, laboratory, and radiological profiles with reference to 40 previously reported overseas cases. Of 699 screened patients, 12 were antibody-positive. Asymmetrical limb ataxia was documented in 75.0% of Japanese patients, whereas asymmetry was explicitly described in only four of twenty-eight overseas patients (14.3%) (p < 0.001); however, this comparison is exploratory and may reflect documentation differences. Japanese patients showed lower cerebrospinal fluid (CSF) inflammatory markers, with lower cell counts (median 4.5/µL vs. 27/µL, p = 0.014) and protein levels (median 36.5 mg/dL vs. 60.0 mg/dL, p = 0.048), although neither remained significant after Bonferroni correction. Two Japanese cases developed neurological symptoms within 1 month of COVID-19 infection. Although all Japanese patients received immunotherapy (corticosteroids in 100%), long-term functional recovery remained suboptimal; among 9 patients followed for ≥ 6 months, only 33.3% achieved functional independence (modified Rankin Scale ≤ 2) at final follow-up. In this first Japanese multicenter case series, asymmetrical limb ataxia was frequently documented and CSF inflammation was often limited. These exploratory findings may generate hypotheses to facilitate earlier diagnosis and treatment, but require cautious interpretation.

**Supplementary Information:**

The online version contains supplementary material available at https://doi.org/10.1007/s12311-026-02094-z.

## Introduction

In the central nervous system, glutamate is one of the primary excitatory neurotransmitters. Metabotropic glutamate receptors (mGluRs) are a large family of G protein-coupled receptors that mediate central and peripheral excitatory neurotransmission [1]. Among them, mGluR1 is predominantly located postsynaptically, with a particularly high concentration in the cerebellum and limbic system [1]. It plays a crucial role in cerebellar motor development and synaptic plasticity [1].

Autoantibodies to mGluR1 have been implicated in autoimmune cerebellar ataxia, a rare but significant immune-mediated neurological disorder primarily characterized by subacute progressive ataxia [2]. Studies have shown that purified anti-mGluR1 immunoglobulin G from affected patients impairs Purkinje cell excitability, plasticity, and survival in mouse cerebellar Sect. [3]. Furthermore, anti-mGluR1 immunoglobulin G injection into the subarachnoid space of normal mice induced reversible ataxia [3], suggesting that anti-mGluR1 antibodies are directly pathogenic.

To date, we are aware of 24 publications that have reported a total of 43 cases of anti-mGluR1 encephalitis [2–25]. Previously, we reported three cases of anti-mGluR1 antibody-associated cerebellar ataxia [23–25]. However, to our knowledge, no case series has been reported from Japan. Therefore, it remains unclear whether the characteristics of this disease in Japanese patients are similar to those reported internationally.

This study aims to characterize the clinical presentation, treatment response, and prognosis of anti-mGluR1 antibody-associated cerebellar ataxia in a Japanese cohort, and to describe these findings with reference to previously reported overseas cases as a literature-based reference group, rather than as a strictly comparable control cohort.

## Patients and Methods

In this retrospective cross-sectional study, we investigated the presence of anti-mGluR1 antibodies in samples collected from patients suspected of having autoimmune cerebellar ataxia, for whom alternative etiologies—such as hereditary, metabolic, toxic, or infectious causes—had been excluded beforehand, and antibody testing was requested to our laboratory specifically for the differential diagnosis of an immune-mediated mechanism. These samples were sent to the Gifu University Graduate School of Medicine upon request from 257 medical institutions in Japan; however, this referral network did not constitute a population-based registry.

Initial screening was performed using a cell-based assay (CBA) with mGluR1-transfected COS7 cells, using patient serum and, when available, cerebrospinal fluid (CSF) samples. For samples demonstrating positivity on the CBA, tissue-based assays (TBA) were subsequently performed on rat brain sections, as previously described [23]. A characteristic neuropil immunostaining pattern consistent with mGluR1 distribution was used as confirmatory evidence. Definite positivity was determined based on concordant CBA and TBA results; when CSF was available, CSF antibody positivity was considered particularly important for diagnostic specificity. To ensure diagnostic objectivity, all assay results were independently evaluated by two investigators (A. Takekoshi and A. Kimura).

The clinical characteristics of Japanese patients who tested positive for anti-mGluR1 antibodies were investigated, including age at onset, sex, prodromal symptoms, comorbidities, clinical manifestations, therapies, and outcomes. Laboratory evaluations included routine CSF analysis, including cell count and protein levels, in all patients, and anti-mGluR1 antibody testing in CSF when CSF samples were available for submission to our laboratory. Neuroimaging assessments included brain magnetic resonance imaging (MRI) and single-photon emission computed tomography (SPECT). Immunotherapy details were obtained for all patients. To evaluate treatment efficacy and long-term prognosis, after the confirmation of antibody positivity, we requested the attending physicians to provide longitudinal clinical data. The patients’ clinical statuses, including the modified Rankin Scale (mRS) and the Scale for the Assessment and Rating of Ataxia (SARA) were tracked and reported by the treating neurologists approximately every 6 months throughout the study period. Treatment outcomes were classified based on the mRS scores. In addition, an exploratory univariable analysis was performed within the Japanese cohort to examine factors associated with functional outcome. Only patients with a clinical follow-up period of at least 6 months were included in this analysis. Patients were classified into a good prognosis group (mRS score ≤ 2 at the last follow-up) and a poor prognosis group (mRS score ≥ 3 at the last follow-up).

After characterizing the clinical features, laboratory findings, treatment approaches, and outcomes of the Japanese cases, these findings were described in relation to a literature-based reference group of previously reported overseas cases. We reviewed the literature to identify reported cases with anti-mGluR1 antibodies published up to March 2025, matching the inclusion period of our Japanese cases. These overseas cases were not re-evaluated using the same cell-based assay (CBA) and tissue-based assay (TBA) methodology or identical inclusion criteria as the Japanese cases. Instead, previously published cases reported as anti-mGluR1-associated disease were included when essential clinical information, including age, sex, and symptoms at onset, was available. Therefore, the overseas cases were analyzed as reported in the original literature. Given the heterogeneous nature of the published case reports, comparative analyses for specific clinical parameters, such as neuroimaging findings or treatment regimens, were conducted using only the available data from cases where those specific details were documented; missing parameters were excluded from the respective analyses. Asymmetrical limb ataxia was defined pragmatically based on clinical documentation rather than a quantitative scale. Japanese cases were classified as having asymmetrical limb ataxia when the treating neurologist explicitly described side predominance of limb ataxia in the medical record during routine neurological examination. No prespecified quantitative threshold or dedicated rating instrument was used for this classification. Cases without an explicit description of laterality were not classified as asymmetrical. For overseas cases, asymmetry was counted only when laterality was explicitly described in the original report. Regarding the chronological analyses for the intervals from symptom onset to CSF collection and immunotherapy initiation, the extracted timeframes included both explicitly stated durations and estimated periods reasonably inferred from the context of the case descriptions. To enable statistical comparisons, these chronological data were uniformly converted into numerical values using months as the standard unit (e.g., within a few days to 0.1 months, approximately 1 week to 0.25 months, and a few weeks to 0.5 months). As a sensitivity analysis, CSF-related variables were re-compared after restricting the dataset to cases in which CSF sampling had been performed within 1 month of symptom onset. For the specific analysis of long-term functional outcomes, however, we applied stricter selection criteria to both Japanese and overseas cases to ensure prognostic reliability. Specifically, only cases with a clearly documented clinical follow-up period of at least 6 months from disease onset were included in the outcome analysis. In addition, overseas cases were required to have explicit and precise reporting of formal mRS or SARA scores at the last follow-up. Cases failing to meet these stringent criteria were excluded from the prognostic comparison to eliminate potential reconstruction bias. Functional independence was defined as a good prognosis (mRS score ≤ 2), whereas an mRS score of ≥ 3 was defined as a poor prognosis [2].

### Statistical Analysis

Continuous variables were expressed as medians [range] and compared using the Mann-Whitney U test. Categorical variables were compared using Fisher’s exact test. A p-value < 0.05 was considered to be statistically significant. The comparison between the Japanese and overseas cohorts was exploratory in nature and was undertaken to describe the clinical characteristics of the Japanese cohort in the context of previously reported cases. Because multiple univariable comparisons were performed, p values were interpreted cautiously. Bonferroni correction was applied to five prespecified comparisons—asymmetrical limb ataxia, CSF cell count, CSF protein level, corticosteroid treatment, and functional independence at the last follow-up (mRS ≤ 2)—with adjusted p values presented accordingly. For the exploratory prognostic analysis within the Japanese cohort, continuous variables were compared using the Mann–Whitney U test and categorical variables using Fisher’s exact test; because of the small sample size, multivariable analysis was not performed. All statistical analyses were performed using R software version 4.1.3. To ensure transparency and reproducibility, the relevant patient-level and comparative data are provided in the main tables and supplementary tables.

## Results

### Clinical Characteristics of Anti-mGluR1 Antibody-Associated Cerebellar Ataxia in Japanese Cases

**Fig. 1:**
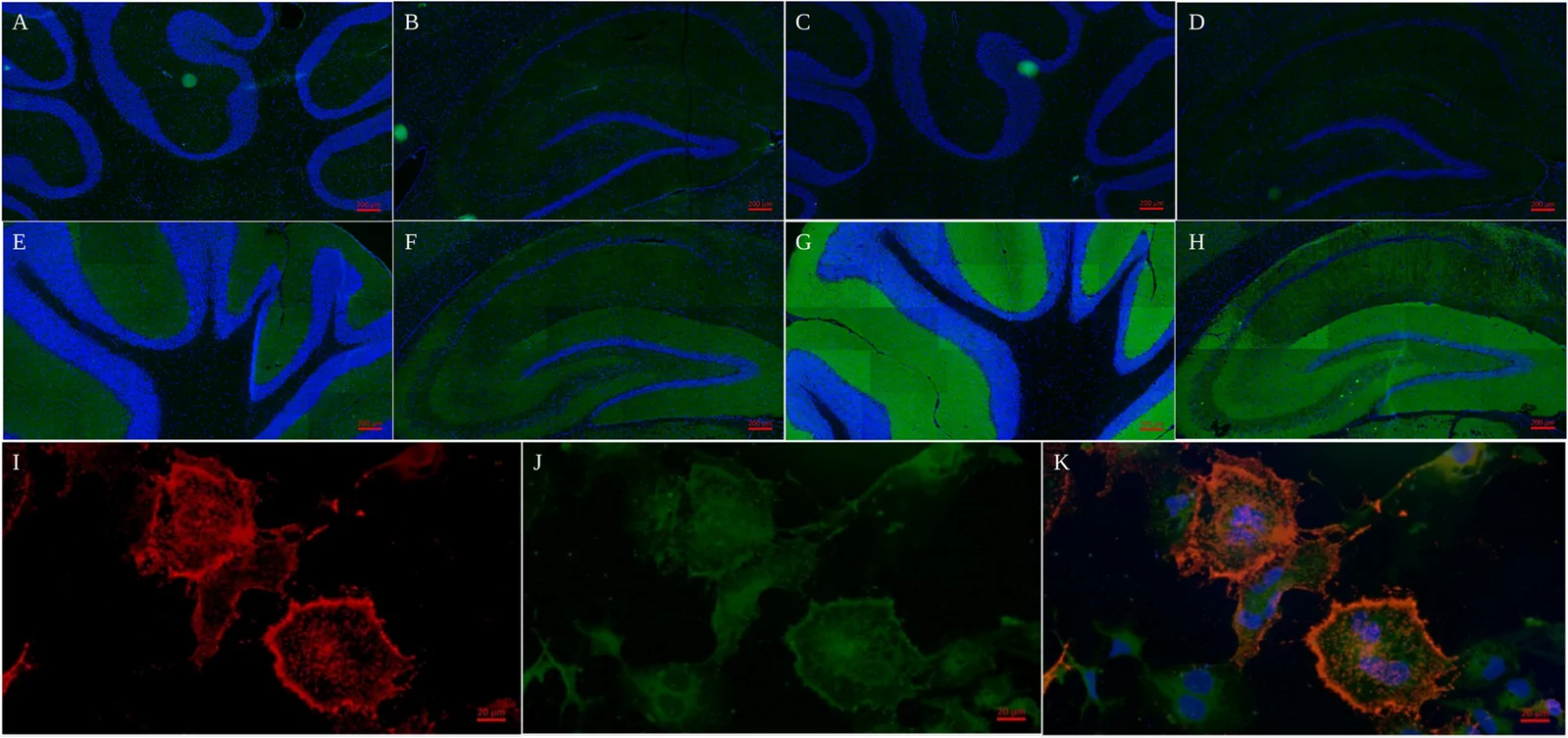
Representative tissue-based assay (TBA) and cell-based assay (CBA) findings in a patient with anti-mGluR1 antibody-associated cerebellar ataxia (**A**, **B**) TBA using serum from a healthy control (1:100) on rat cerebellar and hippocampal sections, respectively (**C**, **D**) TBA using cerebrospinal fluid (CSF) from a disease control patient with idiopathic normal-pressure hydrocephalus (1:4) on rat cerebellar and hippocampal sections, respectively (**E**, **F**) TBA using serum from an anti-mGluR1 antibody-positive patient (1:100) on rat cerebellar and hippocampal sections, respectively (**G**, **H**) TBA using CSF from the same patient (1:4) on rat cerebellar and hippocampal sections, respectively (**I**–**K**) CBA using COS7 cells. (**I**) Patient serum (1:50). (**J**) GFP-labeled cell-surface expression of mGluR1. (**K**) Merged image

During the study period, 699 patients underwent anti-mGluR1 antibody testing, of whom 12 were identified as positive. Anti-mGluR1 antibodies were detected in both serum and CSF in all 12 patients. The median age and range at onset were 54.5 (median) years [5–75] (range), and 6 of 12 patients were women (Table 1). Serum and CSF anti-mGluR1 antibody titers are shown in Table 2. Furthermore, in tissue-based assays using rat brain sections, CSF from all 12 patients demonstrated a characteristic neuropil immunostaining pattern, predominantly in the cerebellar molecular layer, consistent with the expected mGluR1 distribution. Representative TBA and CBA findings are shown in Fig. 1.

**Table 1:** Patient demographics

Characteristics	Results
Age	
Median (years) [range]	54.5 [5–75]
< 10 years, n (%)	1 (8.3%)
40–49 years, n (%)	1 (8.3%)
50–59 years, n (%)	5 (41.7%)
60–69 years, n (%)	2 (16.7%)
70–79 years, n (%)	3 (25%)
sex, male/female	6/6

Clinical data for three Japanese patients included in this series were previously reported [23–25]

**Table 2:** Summary of the clinical features, investigations, treatment, and outcomes of Japanese patients with anti-mGluR1 antibody-associated cerebellar ataxia

Pt.	Associated condition(s)	Major clinical symptoms and signs	Anti-mGluR1 Ab titer (serum/CSF)	CSF findings (cells, protein, OCB)	MRI findings (initial/follow-up)	Interval from onset to CSF/treatment (mo.)	Immunotherapy	mRS/SARA (presentation, 6 mo., last follow-up)
1*	−	Cerebellar ataxia	≥ 1:3200 / 1:16	5, 29, +	normal/cerebellar atrophy	2/3	IVMP, oral PSL, IVIG, PP, TAC→AZA (discontinued), RTX	4/15, 3/14, 3/12
2	Ulcerative colitis	Cerebellar ataxia	≥ 1:3200 / ≥1:32	6, 22, −	normal/normal	0.75/1	IVMP, IVIG, TAC (discontinued)	2/8, 1/0, 1/0
3	−	Cerebellar ataxia	≥ 1:3200 / ≥1:32	7, 60, +	normal/cerebellar atrophy	0.25/1	IVMP, oral PSL, PP	4/18.5, 4/N/A, 3/15
4*	−	Cerebellar ataxia	1:800 / 1:4	1, 31, −	normal/N/A	4.5/6	IVMP, IVIG	4/19, 0/0, 0/0
5	−	Cerebellar ataxia	≥ 1:3200 / ≥1:32	1, 37, −	normal/CT: cerebellar atrophy	2/3	IVMP, oral PSL, IVIG	2/N/A, 4/N/A, 3/22
6	−	Cerebellar ataxia with ocular motor features	≥ 1:3200 / ≥1:32	4, 26, +	normal/N/A	1/1	oral PSL, PP	4/19, 3/13, 3/11.5
7*	−	Cerebellar ataxia	1:200 / 1:16	4, 40, +	normal/N/A	3/3	IVMP, oral PSL, IVIG, RTX	4/24, 1/9, 1/9
8	COVID-19; lung adenocarcinoma; autoimmune hepatitis	Cerebellar ataxia with cognitive/sensory features	≥ 1:3200 / 1:2	1, 31, +	FLAIR hyperintensity in right occipital lobe/cerebellar atrophy	12/2	IVMP, oral PSL, IVIG	4/32, 4/30.5, 4/30.5
9	COVID-19	Cerebellar ataxia with cognitive features	≥ 1:3200 / ≥1:32	7, 59, −	cerebellar atrophy/N/A	12/12	IVMP, oral PSL, IVIG, TAC	4/13, 4/14.5, 4/14.5
10	−	Cerebellar ataxia	1:800 / ≥1:32	4, 53, −	normal/N/A	3/3	IVMP	4/N/A, N/A/N/A, N/A/N/A
11	Gastroenteritis	Cerebellar ataxia	1:400 / 1:16	69, 36, +	high FLAIR signal in bilateral cerebellar cortices/improved	0.25/0.75	IVMP, oral PSL	2/N/A, N/A/N/A, N/A/N/A
12	Lung carcinoma	Cerebellar ataxia with cognitive/psychiatric features	≥ 1:3200 / ≥1:32	87, 337, +	normal/N/A	0.25/0.25	IVMP, oral PSL, IVIG	3/N/A, N/A/N/A, N/A/N/A

*CSF* cerebrospinal fluid, *CT* computed tomography, *FLAIR* fluid-attenuated inversion recovery, *mGluR* metabotropic glutamate receptor, *mRS* modified Rankin Scale, *N/A* not available, *IVIG* intravenous immunoglobulin, *IS* immunosuppressive agents, *IVMP* intravenous methylprednisolone, *OCB* oligoclonal band, *PP* plasmapheresis, *PSL* prednisolone, *RTX* rituximab, *SARA* Scale for the Assessment and Rating of Ataxia. Anti-mGluR1 antibody titers were determined by cell-based assay and are expressed as the highest serum and CSF dilution showing a positive result; tissue-based assay was used for confirmation of antibody positivity. Values shown as “≥1:3200” (serum) or “≥1:32” (CSF) indicate that the samples remained positive at the maximum dilution tested; exact endpoint titers were not determined because higher dilutions were not examined. Patients marked with an asterisk (*) were previously reported [23–25]

Progressive truncal ataxia leading to gait disturbance was the major clinical presentation in 11 patients. The remaining patient initially presented with spatial disorientation and paresthesia in the legs, after which cerebellar ataxia became the leading clinical manifestation several months later. The median time from onset to admission was 2.0 months [0.25–11], while that from onset to sample collection was 2.0 months [0.25–12]. Regarding comorbidities, one patient developed ulcerative colitis, while another was diagnosed with autoimmune hepatitis. Two patients had lung carcinoma. A prior infection was identified in three patients (COVID-19 in two, gastroenteritis in one) (Table 2).

During the disease course, cerebellar ataxia was the predominant clinical syndrome in all patients. All patients exhibited both truncal and limb ataxia during the disease course. Dysarthria was observed in ten patients, nystagmus in eight, and titubation in two. In addition, nine patients displayed asymmetric limb ataxia as determined through clinical assessment made by their attending physicians (Supplementary Table 1).

Extracerebellar symptoms were observed in nine patients during their clinical course, most commonly dysgeusia in four and cognitive impairment in three. Other extracerebellar manifestations included myokymia in one patient and diplopia due to bilateral abducens nerve palsy in one. Detailed extracerebellar manifestations are summarized in Table 2 and Supplementary Table 1.

In the initial CSF analysis, the median cell count was 4.5/µL [1–87] and the protein level 36.5 mg/dL [22–337]. Oligoclonal bands were detected in seven cases (Table 2).

**Fig. 2:**
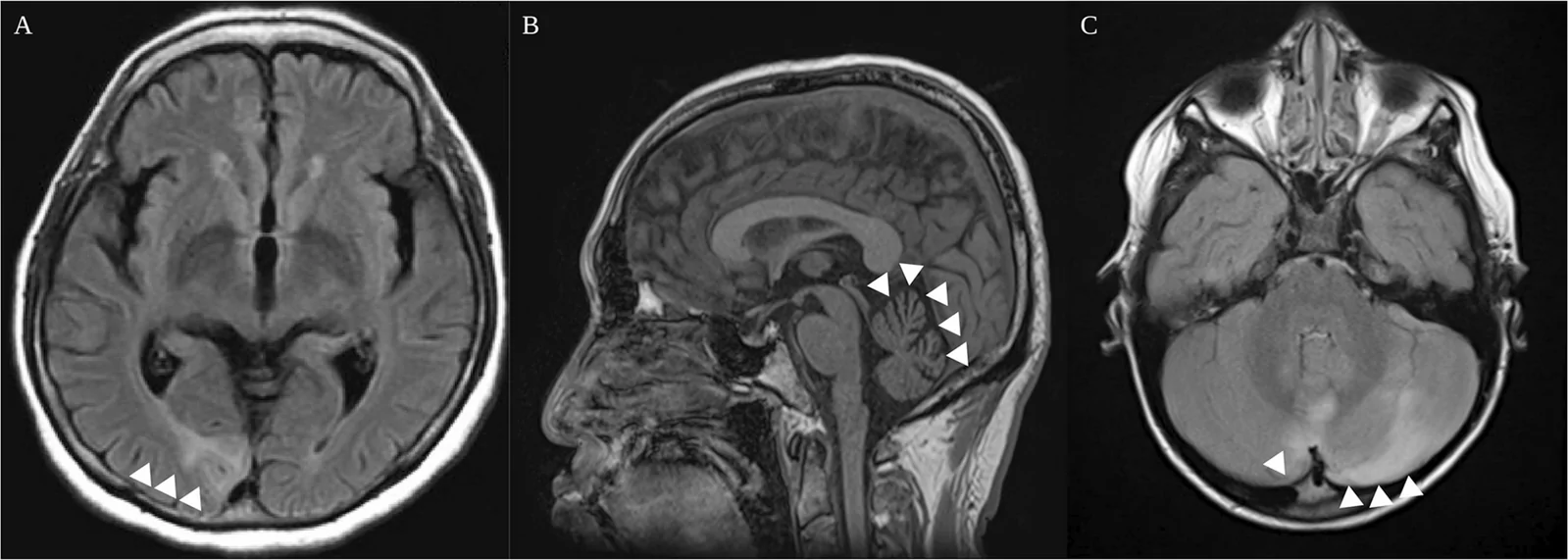
Representative MRI findings in Japanese patients with anti-mGluR1 antibody-associated cerebellar ataxia (A) Fluid-attenuated inversion recovery (FLAIR) MRI showing a hyperintense lesion (white arrowheads) in the right occipital lobe (Pt. 8) (B) Sagittal T1-weighted brain MRI showing cerebellar atrophy (white arrowheads) (Pt. 9) (C) FLAIR MRI showing hyperintense lesions (white arrowheads) in the cerebellar vermis and bilateral cerebellar hemispheres, with left-sided predominance (Pt. 11)

Brain MRI was initially normal in nine patients. Of the remaining three patients, the first exhibited a fluid-attenuated inversion recovery hyperintensity lesion in the right occipital lobe, the second displayed cerebellar atrophy, and the third showed fluid-attenuated inversion recovery hyperintensity in the bilateral cerebellar cortices (Fig. 2).

Follow-up imaging studies were available in six patients (MRI, *n* = 5; CT, *n* = 1). Cerebellar atrophy was observed in three patients on follow-up MRI and in one patient on follow-up CT (Table 2). SPECT analysis was performed in ten patients. Normal findings were observed in eight cases, whereas one patient exhibited decreased blood flow in the right cerebellum (Pt. 6) and another displayed bilateral cerebellar hemispheric hypoperfusion with right-side predominance (Pt. 7).

All patients received immunotherapy. The duration from disease onset to the initiation of first immunotherapy was 2.5 months [0.25–12]. Treatment regimens included methylprednisolone pulse therapy in 11 patients, oral prednisolone in 9, intravenous immunoglobulin in 8, plasmapheresis in 3, rituximab in 2, and immunosuppressants in 3: the first of whom switched from tacrolimus to azathioprine, both of which were eventually discontinued due to multiple relapses, and received long-term repeated cycles of rituximab; the second received tacrolimus but later stopped; and the third received tacrolimus for maintenance therapy (Table 2).

At the time of sampling, the mRS score was 4 [2–4] and the SARA score 18.75 [8–32]. At the final follow-up, among the nine patients who had been followed for more than 6 months, the mRS score was 3 [0–4] and the SARA score 12.0 [0–30.5]. Following immunotherapy, three patients achieved an mRS score of ≤ 2 at the final follow-up. Two patients (Pt. 1 and Pt. 8) experienced clinical relapses during their courses (Table 2, Supplementary Table 2). An exploratory univariable analysis was additionally performed within the Japanese cohort according to functional outcome at the last follow-up. Three patients were classified into the good prognosis group (mRS score ≤ 2) and six into the poor prognosis group (mRS score ≥ 3). Comparisons of demographic variables, CSF findings, treatment-related variables, and neurological severity at presentation did not identify any clear baseline factors associated with prognosis. Because of the small sample size, these findings should be interpreted cautiously as exploratory. Detailed results are shown in Supplementary Table 3.

### Comparisons of Patients with Anti-mGluR1 Antibody-Associated Cerebellar Ataxia Between Japanese and Overseas Cases (Table 3)

**Table 3:** Comparison of clinical findings between Japanese and overseas cases

Category	Variable	Japanese cases (*N* = 12)	Overseas cases (*N* = 40)	*p* value	95% CI	Adjusted *p* value	Adjusted CI (99% CI)
Patient Demographics	Age (years) median [range]	54.5 [5–75]	50.0 [2–81]	0.142	[-3.00, 25.00]	–	–
	Sex (female, %)	6 (50.0%)	18 (45.0%)	> 0.999	[0.27, 5.45]	–	–
	Pediatric patients, n (%)	1 (8.3%)	5 (12.5%)	> 0.999	[0.01, 6.70]	–	–
	Prior infection, n (%)	3 (25.0%)	11 (27.5%)	> 0.999	[0.13, 4.48]	–	–
	Weight loss, n (%)	0	3 (7.5%)	> 0.999	–	–	–
	Malignancy, n (%)	2 (16.7%)	8 (21.1%), *n* = 38	> 0.999	[0.07, 4.76]	–	–
	Autoimmune diseases, n (%)	2 (16.7%)	8 (20.5%), *n* = 39	> 0.999	[0.07, 4.91]	–	–
Symptoms and signs	Cerebellar ataxia, n (%)	12 (100%)	38 (95.0%)	> 0.999	–	–	–
	Truncal ataxia, n (%)	12 (100%)	36 (94.7%), *n* = 38	> 0.999	–	–	–
	Limb ataxia, n (%)	12 (100%)	28 (73.7%), *n* = 38	0.092	–	–	–
	Asymmetrical limb ataxia, n (%)	9 (75.0%)	4 (14.3%), *n* = 28	< 0.001	[2.68, 137.80]	0.002	[1.97, 164.08]
	Dysarthria, n (%)	10 (83.3%)	24 (63.2%), *n* = 38	0.292	[0.50, 30.57]	–	–
	Titubation, n (%)	2 (16.7%)	6 (15.8%), *n* = 38	> 0.999	[0.09, 7.32]	–	–
	Extracerebellar symptoms and signs, n (%)	9 (75.0%)	27 (67.5%)	0.733	[0.29, 9.62]	–	–
	Psychiatric symptoms, n (%)^a^	1 (8.3%)	11 (27.5%)	0.253	[0.01, 2.08]	–	–
	Cognitive impairment, n (%)	3 (25.0%)	9 (22.5%)	> 0.999	[0.22, 8.77]	–	–
	Seizure, n (%)	0	4 (10.0%)	0.562	–	–	–
	Other movement disorder, n (%)^b^	1 (8.3%)	4 (10.0%)	> 0.999	[0.01, 6.70]	–	–
	Dysgeusia, n (%)	4 (33.3%)	5 (12.5%)	0.185	[0.55, 20.27]	–	–
	Sleep disorder, n (%)	0	2 (5.0%)	> 0.999	–	–	–
CSF findings	Duration from onset to CSF sampling (months)	2.0 [0.25–12]	1.0 [0.1–84], *n* = 23	0.649	[-0.75, 2.00]	–	–
	Cell counts (/µL)	4.5 [1–87]	27.0 [2–240], *n* = 21	0.014	[-49.00, -2.00]	0.070	[-125.5, 10.5]
	Protein levels (mg/dL)	36.5 [22–337]	60.0 [28–85], *n* = 10	0.048	[-38.40, 0.00]	0.240	[-45.2, 9.0]
	Oligoclonal band (positive, %)	7 (58.3%)	14 (58.3%), *n* = 24	> 0.999	[0.20, 5.27]	–	–
	Cell counts (/µL) within 1month of symptom onset	7.0 [4–87], *n* = 5	28 [4–240], *n* = 12	0.245	[-183.00, 42.00]	–	–
	Protein levels (mg/dL) within 1month of symptom onset	36 [22–337], *n* = 5	54 [28–77], *n* = 5	0.548	[-50.00, 283.00]	–	–
MRI findings							
initial MRI	Normal findings, n (%)	9 (75.0%)	18 (51.4%), *n* = 35	0.191	[0.05, 1.78]	–	–
	T2/FLAIR hyperintensity in cerebellum, n (%)	1 (8.3%)	9 (25.7%), *n* = 35	0.413	[0.06, 2.38]	–	–
	Cerebellar atrophy, n (%)	1 (8.3%)	3 (8.6%), *n* = 35	> 0.999	[0.02, 13.62]	–	–
	Asymmetrical cerebellar lesion, n (%)	1 (8.3%)	3 (8.6%), *n* = 35	> 0.999	[0.02, 13.62]	–	–
	Other abnormal findings, n (%)^c^	1 (8.3%)	5 (14.3%), *n* = 35	> 0.999	[0.02, 5.79]	–	–
follow-up MRI	Cerebellar atrophy, n (%)	3 (60.0%), *n* = 5	10 (71.4%), *n* = 14	> 0.999	[0.05, 10.06]	–	–
Treatment	Duration from onset to treatment (months)	2.5 [0.25–12]	1.0 [0.25–36], *n* = 23	0.738	[-2.00, 2.00]	–	–
	First-line therapy, n (%)	12 (100%)	32 (84.2%), *n* = 38	0.314	–	–	–
	Corticosteroid (IVMP, oral PSL), n (%)	12 (100%)	26 (68.4%), *n* = 38	0.047	–	0.235	–
	IVIG, n (%)	8 (66.7%)	22 (57.9%), *n* = 38	0.740	[0.32, 7.73]	–	–
	PP, n (%)	3 (25.0%)	5 (13.2%), *n* = 38	0.379	[0.28, 13.83]	–	–
	Second-line therapy, n (%)	4 (33.3%)	17 (44.7%), *n* = 38	0.526	[0.12, 2.83]	–	–
	IS, n (%)	3 (25.0%)	7 (18.4%), *n* = 38	0.686	[0.20, 8.27]	–	–
	Rituximab, n (%)	2 (16.7%)	11 (28.9%), *n* = 38	0.480	[0.05, 2.95]	–	–
	Cyclophosphamide, n (%)	0	5 (13.2%), *n* = 38	0.319	–	–	–
Disease severity and outcome	mRS at the presentation	4.0 [2–4]	4.0 [1–5], *n* = 13	0.906	[-1.00, 1.00]	–	–
	SARA at the presentation	18.75 [8–32], *n* = 8	18 [0–36], *n* = 13	0.856	[-10.00, 1.50]	–	–
	Follow-up period (cases included in outcome analysis), months	24.0 [6–132], *n* = 9	41.0 [6–168], *n* = 16	0.379	[-45.00, 12.00]	–	–
	mRS at last follow-up^d^	3.0 [0–4], *n* = 9	2.0 [0–6], *n* = 14	0.460	[-1.00, 2.00]	–	–
	SARA at last follow-up^d^	12.0 [0–30.5], *n* = 9	9.0 [0–36], *n* = 11	0.566	[-9.00, 14.5]	–	–
	mRS ≤ 2 at last follow-up, n (%)^d^	3 (33.3%), *n* = 9	10 (71.4%), *n* = 14	0.102	[0.03, 1.22]	0.512	[0.02, 2.15]
	Relapse, n (%)	2 (22.2%), *n* = 9	8 (20.0%)	> 0.999	[0.11, 9.50]	–	–

^a^ Psychiatric symptoms include hallucinations, delusions, apathy, catatonia, and behavioral changes

^b^ Other movement disorder includes myokymia in the right leg in one Japanese patient and myoclonus in two (2, 11), chorea in one (2) and slowness in writing in one (2) in overseas cases

^c^ Other abnormal findings include a right occipital FLAIR hyperintense lesion in one Japanese patient (Pt. 8), subcortical dot lesions in the frontal lobe in one (2), multiple brain and spinal cord lesions in two (8), a left thalamus lesion in one (10) and a subcortical lesion in the occipital lobe in one (17). The MRI categories are not mutually exclusive; a single patient may be counted in more than one category. Asymmetrical cerebellar lesion was defined as a left–right asymmetry of the cerebellar abnormality

^d^ Long-term outcomes (mRS and SARA scores) and the occurrence of relapses were evaluated exclusively in patients with a clinical follow-up period of 6 months or longer

*FLAIR* fluid attenuated inversion recovery, *mRS* modified Rankin scale, *IVMP* intravenous methyl prednisolone therapy, *IVIG* intravenous immunoglobulin, *IS* immunosuppressants, *PP* plasmapheresis, *PSL* prednisolone, *RTX* rituximab, *SARA* Scale for the Assessment and Rating of Ataxia

Continuous variables are presented as median [range], and confidence intervals indicate the interval for the between-group difference. Categorical variables are presented as n (%), and confidence intervals indicate the interval for the odds ratio. For the five key comparisons, Bonferroni-adjusted P values and 99% CIs are shown. Confidence intervals were not estimated when one cell contained zero

In an exploratory comparison of the Japanese cohort (*N* = 12) with the literature-based overseas reference cases (*N* = 40), no significant differences were observed in age at onset, sex, the frequency of prior infections, coexisting other autoimmune diseases, or malignancies. Regarding the cerebellar ataxia, asymmetrical limb ataxia was documented more frequently in Japanese cases than in overseas cases (9/12 [75.0%] vs. 4/28 [14.3%], *p* < 0.001). The overall proportion of patients who developed extracerebellar symptoms during the disease course did not differ significantly between the two groups (9 (75.0%) vs. 27 (67.5%), *p* = 0.733).

Regarding CSF findings, the interval from symptom onset to CSF collection was 2.0 months [0.25–12] in the Japanese cohort and 1.0 month [0.1–84] in the overseas cohort, with no significant difference between the two groups (*p* = 0.649). In the overall comparison, Japanese patients showed lower cell counts (median 4.5/µL [1–87] vs. 27/µL [2–240], *p* = 0.014) and protein levels (36.5 mg/dL [22–337] vs. 60 mg/dL [28–85], *p* = 0.048) than overseas patients; however, these differences did not remain significant after Bonferroni correction. The rate of oligoclonal band positivity was identical between the two groups, occurring in 7 of 12 (58.3%) Japanese patients and 14 of 24 (58.3%) overseas patients (*p* > 0.999). In a sensitivity analysis restricted to cases in which CSF sampling was performed within 1 month of symptom onset, the between-cohort differences in CSF cell count and protein level were not confirmed.

There was no significant difference between the Japanese (3/12 [25%]) and overseas (17/35 [48.6%]) groups in the proportion of any abnormal findings on initial MRI (*p* = 0.191). Follow-up MRI revealed cerebellar atrophy in a high proportion of both groups, occurring in 3 of 5 (60.0%) Japanese patients and 10 of 14 (71.4%) overseas patients (*p* > 0.999).

Regarding treatment, the interval from onset to the initiation of immunotherapy tended to be longer in Japanese patients, but the difference was not statistically significant (median 2.5 vs. 1.0 months, *p* = 0.738). The proportion of patients receiving first-line therapy (12 (100%) vs. 32 (84.2%), *p* = 0.314) and second-line therapy (4 (33.3%) vs. 17 (44.7%), *p* = 0.526) did not differ significantly between Japanese patients and overseas patients. Corticosteroid therapy was used more frequently in Japanese patients than in overseas patients (12 (100%) vs. 26 (68.4%), *p* = 0.047), although this difference did not remain significant after Bonferroni correction. Additionally, the use of cyclophosphamide as a second-line treatment was reported only in overseas cases.

No significant differences were observed between Japanese and overseas patients in pre-treatment mRS scores (4.0 [2–4], *n* = 12 vs. 4.0 [1–5], *n* = 13, *p* = 0.906) or SARA (18.75 [8–32], *n* = 8 vs. 18 [0–36], *n* = 13, *p* = 0.856). Regarding long-term clinical outcomes evaluated in patients followed for 6 months or longer, the follow-up duration did not differ significantly between the two groups (24.0 [6–132] months for Japanese patients [*n* = 9] vs. 41.0 [6–168] months for overseas patients [*n* = 16], *p* = 0.379). Japanese patients tended to show higher mRS scores at last follow-up (3 [0–4], *n* = 9 vs. 2.0 [0–6], *n* = 14, *p* = 0.460), higher SARA scores at last follow-up (12.0 [0–30.5], *n* = 9 vs. 9.0 [0–36], *n* = 11, *p* = 0.566), and a lower proportion achieving functional independence (mRS score ≤ 2) at last follow-up (3 of 9 [33.3%] vs. 10 of 14 [71.4%], *p* = 0.102); however, none of these differences reached statistical significance.

## Discussion

To our knowledge, this represents the first systematically collected multicenter case series from Japan. On the basis of our findings, we discuss four observations regarding this disease.

First, in the overall comparison, Japanese patients tended to show lower CSF cell counts and protein levels than literature-based overseas reference cases, whereas the frequency of oligoclonal band positivity was similar between the two groups. This observation may be of clinical interest because anti-mGluR1 antibody-associated cerebellar ataxia can present with only modest abnormalities in routine CSF inflammatory markers despite clinically significant cerebellar dysfunction. At the same time, this observation should be interpreted carefully. Differences in the timing of lumbar puncture may have contributed to the observed between-cohort differences. The median duration from symptom onset to CSF sampling was 2.0 months [0.25–12] in the Japanese cohort and 1.0 month [0.1–84] in the overseas cohort (*p* = 0.649). Although this difference was not statistically significant, the wide range in the overseas cohort likely reflects a heterogeneous population with variable timing of evaluation. In addition, all Japanese cases were identified during a relatively recent period (2018–2025), whereas the literature-based overseas reference cases were derived from reports published across a broader historical range. Therefore, we cannot exclude the possibility that differences in sampling timing, referral pathways, and the level of detail reported in published cases may have influenced the observed CSF profiles. Moreover, in the sensitivity analysis restricted to cases in which CSF sampling was performed within 1 month of symptom onset, the between-cohort differences in CSF cell count and protein level were not confirmed. Taken together, the observed CSF differences should be interpreted as exploratory rather than definitive evidence of a biological regional difference. Notably, despite the apparently milder routine CSF inflammatory profile, the rate of cerebellar atrophy on follow-up MRI remained high and comparable between the Japanese and overseas cohorts. This finding is clinically important because a near-normal CSF profile should neither exclude the diagnosis of anti-mGluR1 antibody-associated cerebellar ataxia nor be assumed to predict a benign structural outcome. In this context, the similar frequency of oligoclonal band positivity between the two groups suggests that intrathecal immune activation may still be present even when routine CSF inflammatory markers are modest. Further studies with standardized CSF assessment in larger cohorts will be needed to determine whether these findings reflect differences in sampling timing and case ascertainment, true biological variation, or both.

Second, asymmetrical limb ataxia was documented in 9 of 12 patients (75.0%) in our cohort. In literature-based overseas reference cases, asymmetry was explicitly described in only 4 of 28 published cases with limb ataxia or a pancerebellar phenotype (14.3%; *p* < 0.001). However, this difference should be interpreted cautiously because the overseas comparator was derived from published reports with heterogeneous levels of neurologic detail, and asymmetry was counted only when explicitly stated. Moreover, although the higher frequency of asymmetrical limb ataxia in the Japanese cohort remained statistically significant after Bonferroni correction (adjusted *p* = 0.002), the 99% confidence interval for the odds ratio was very wide (1.97–164.08), indicating substantial imprecision in the magnitude of the effect estimate. This finding should therefore be regarded as hypothesis-generating rather than as evidence of a definitive regional characteristic. The lack of a description of asymmetry should not be interpreted as evidence of symmetry. Asymmetrical limb ataxia is not unique to anti-mGluR1 antibody-associated cerebellar ataxia and has also been reported in other autoimmune cerebellar syndromes [26, 27]. Therefore, asymmetry should not be used to narrow antibody testing to a limited set of targets, but rather to prompt consideration of a broad autoimmune differential diagnosis in patients with subacute cerebellar ataxia.

Third, corticosteroids appeared to be used more frequently in the Japanese cohort than in literature-based overseas reference cases; however, this difference did not remain significant after Bonferroni correction and should therefore be interpreted cautiously. This difference may partly reflect an era effect, as many overseas cases were reported between 2000 and 2018, when the clinical recognition of this rare disorder and the treatment framework for autoimmune encephalitis were less standardized. In contrast, all Japanese cases were evaluated between 2018 and 2025, during a more recent period in which structured immunotherapy-based treatment approaches for autoimmune encephalitis had become more widely integrated into clinical practice [28, 29]. This chronological difference may have contributed to the more systematic use of corticosteroids in the Japanese cohort. However, the median interval from symptom onset to treatment initiation was 2.5 months [0.25–12] in the Japanese cohort and 1.0 month [0.25–36] in the overseas cohort (*p* = 0.738), indicating that treatment was not initiated earlier in the Japanese cohort. Although this difference was not statistically significant, the numerically longer interval in the Japanese cohort may in part reflect delayed recognition of autoimmune cerebellar ataxia in routine practice and/or longer referral pathways before patients reach centers capable of specialized antibody testing and neuroimmunological evaluation. Differences in referral pathways and case ascertainment may also have influenced the observed treatment patterns. Notably, despite immunotherapy, residual disability and cerebellar atrophy remained common in the Japanese cohort, and long-term outcomes were not significantly different from those of previously reported overseas cases. However, the long-term outcome analyses were based on a limited and uneven subset of cases, because sufficient follow-up data (≥ 6 months) were available for only a minority of the overseas cohort, and the denominators differed across outcome measures. Accordingly, no strong inference should be drawn from these incomplete comparisons regarding potential differences in prognosis between the cohorts. Taken together, these findings suggest that incomplete recovery remains an important clinical issue in anti-mGluR1 antibody-associated cerebellar ataxia despite immunotherapy. In an additional exploratory univariable analysis within the Japanese cohort, no clear baseline clinical, CSF, or treatment-related factor was identified as a robust predictor of functional outcome. However, this result should be interpreted cautiously because of the very small sample size and heterogeneity in treatment and follow-up. Further accumulation of cases will be necessary to clarify whether prognosis is influenced by baseline disease severity, diagnostic delay, or differences in immunotherapy.

Fourth, two patients in our cohort developed neurological symptoms within 1 month of COVID-19 infection, indicating a temporal association. This observation raises the possibility that COVID-19-related immune activation may have acted as a trigger; however, no causal inference can be made from the present data. Previous reports have described antecedent infections, including herpes zoster, dengue fever, and flu-like illnesses, in some patients with anti-mGluR1 antibody-associated cerebellar ataxia [2, 7, 10, 12]. At the same time, antecedent infection history was not systematically reported in many previously published overseas cases, and many of these cases were reported before the COVID-19 pandemic. Therefore, the apparent absence of similar post-COVID-19 cases in earlier reports should be interpreted cautiously. These observations should be regarded as hypothesis-generating and require confirmation in larger cohorts.

Our study has several limitations. First, the sample size was small, reflecting the rarity of anti-mGluR1 antibody-associated cerebellar ataxia. Second, the Japanese cohort was assembled through a referral-based laboratory network and was enriched for patients with suspected autoimmune cerebellar ataxia. This referral pattern may have introduced selection bias in both phenotype and disease course, and the cohort may not fully represent the entire clinical spectrum of anti-mGluR1 antibody-associated cerebellar ataxia in Japan. Furthermore, because this was not a population-based registry, some Japanese cases may not have been captured, including cases evaluated outside our referral network or referred to other laboratories. Third, asymmetrical limb ataxia in the Japanese cohort was identified on the basis of explicit documentation by the treating neurologist during routine clinical examination and was not defined using a prespecified quantitative instrument or cutoff. Therefore, inter-rater variability and documentation bias cannot be excluded. Fourth, the study was retrospective, and follow-up duration and the availability of longitudinal clinical data varied across institutions, which may have affected the consistency of outcome assessment. Fifth, the comparison with overseas cases was literature-based and therefore subject to heterogeneous reporting, missing data, and variable follow-up. This limitation is particularly relevant to detailed clinical features such as asymmetrical cerebellar signs, because such information was systematically requested in our cohort but was not uniformly described in published overseas reports; taken together with the documentation-based ascertainment described above, the observed difference in asymmetry between the Japanese and overseas cohorts should be interpreted cautiously. Sixth, although detailed clinical information was available for the Japanese antibody-positive cases, not all clinical and imaging findings in these patients can necessarily be attributed to the anti-mGluR1 antibody–associated disease. For example, in Pt. 8, cognitive impairment and homonymous hemianopia associated with a right occipital FLAIR lesion preceded the onset of cerebellar ataxia by several months, and both the symptoms and the lesion resolved after corticosteroid therapy, suggesting an immune-mediated process; however, anti-mGluR1 antibody testing was performed only after the onset of ataxia, and an occipital lesion with cognitive impairment is not a typical presentation of this disease. Therefore, a definitive attribution of this extracerebellar lesion to the anti-mGluR1 antibody–associated disease could not be made. Similar uncertainty applies to other extracerebellar clinical and imaging findings in our cohort, including psychiatric symptoms and other movement disorders, which should therefore be interpreted with caution. Finally, antibody testing methods were not fully uniform across the Japanese and overseas cases. In our cohort, all patients were evaluated using both serum and CSF, with complementary cell-based and tissue-based assays, whereas the historical overseas reports varied in assay platform, sample type, dilution thresholds, and the availability of confirmatory testing. In addition, the overseas cases were not re-evaluated using the same CBA/TBA methodology or identical inclusion criteria as the Japanese cohort, but were analyzed according to the diagnostic assessments reported in the original publications. Therefore, differences in diagnostic workup and antibody testing methodology may have contributed to case ascertainment bias and limited direct comparability between the Japanese and overseas cohorts.

## Conclusion

This Japanese case series adds to the clinical characterization of anti-mGluR1 antibody-associated cerebellar ataxia. Asymmetrical limb ataxia was frequently documented in our cohort, and routine CSF inflammatory findings tended to be mild; however, these observations are exploratory and should be interpreted cautiously, particularly in light of potential documentation bias and the limited comparability with literature-based overseas cases. Within these limitations, the diagnosis should not be dismissed in patients with subacute cerebellar ataxia simply because CSF abnormalities are limited. Two cases showed temporal association with COVID-19, but the significance of this finding remains uncertain. Persistent disability and cerebellar atrophy were common despite immunotherapy, underscoring the need for larger prospective studies using standardized assessments.

## Supplementary Information

Below is the link to the electronic supplementary material.

Supplementary Material 1

## Data Availability

Anonymized data not included in this article will be made available upon reasonable request from qualified investigators.
