# Supplementary material for: Clinical Characteristics of Japanese Cases of Anti-Metabotropic Glutamate Receptor Type 1 Antibody-Associated Cerebellar Ataxia
Source: Cerebellum. 2026 Sep 30;25(5):148. doi: 10.1007/s12311-026-02094-z (PMC13627236; doi:10.1007/s12311-026-02094-z)
Supplement: Supplementary file 1 [file 12311_2026_2094_MOESM1_ESM.docx]

Supplementary Table 1. Detailed clinical characteristics at presentation of Japanese patients with anti-mGluR1 antibody-associated cerebellar ataxia.

| Pt. | Main initial symptoms at presentation | Onset to presentation  (mo.) | Truncal ataxia | Limb ataxia (dominant site) | Dysarthria | Nystagmus | Titubation | Extracerebellar signs and symptoms (timing of onset) |
| --- | --- | --- | --- | --- | --- | --- | --- | --- |
| 1* | gait disturbance | 2 | + | +, L | + | + | - | dysgeusia (concurrent with cerebellar ataxia) |
| 2 | gait disturbance | 0.75 | + | +, L | + | - | - | dysgeusia (concurrent with cerebellar ataxia) |
| 3 | gait disturbance | 0.25 | + | +, L | + | - | - | myokymia in the right leg (concurrent with cerebellar ataxia) |
| 4* | gait disturbance | 5 | + | + | - | + | + | - |
| 5 | gait disturbance, dysarthria, vertigo | 2 | + | +, L | + | + | - | - |
| 6 | gait disturbance | 1 | + | +, L | + | + | - | diplopia (concurrent with cerebellar ataxia) |
| 7* | gait disturbance, dizziness | 3 | + | +, R | + | - | - | dysgeusia (concurrent with cerebellar ataxia) |
| 8 | paresthesia in legs, spatial disorientation | 9 | + | +, R | + | + | + | cognitive impairment, left homonymous hemianopia, numbness in the lower limbs, decreased vibration sense in the lower limbs (prior to cerebellar ataxia) |
| 9 | gait disturbance | 11 | + | + | + | + | - | cognitive impairment, hyperreflexia (concurrent with cerebellar ataxia) |
| 10 | gait disturbance, dysarthria, vertigo | 3 | + | +, L | + | - | - | dysgeusia (prior to cerebellar ataxia) |
| 11 | headache, gait disturbance, dysarthria | 0.75 | + | +, R | + | + | - | headache (prior to cerebellar ataxia) |
| 12 | hallucination, memory impairment, truncal ataxia | 0.25 | + | + | - | + | - | hallucination, memory deficit (concurrent with cerebellar ataxia) |

Patients marked with an asterisk (*) were previously reported [23–25].

Supplementary Table 2. Detailed neurological symptoms and signs during the disease course in Japanese patients with anti-mGluR1 antibody-associated cerebellar ataxia

| Pt. | mRS and SARA score (mRS/SARA score) | | | | | | Relapse |
| --- | --- | --- | --- | --- | --- | --- | --- |
|  | At sampling | 6M | 12M | 18M | 24M | At last follow-up (duration, months) |  |
| 1* | 4/15 | 3/14 | 3/11 | 3/13.5 | 3/10 | 3/12 (132) | + |
| 2 | 2/8 | 1/0 | 1/0 | 1/0 | 1/0 | 1/0 (70) | - |
| 3 | 4/18.5 | 4/N/A | 4/N/A | 4/N/A | 4/N/A | 3/15 (44) | - |
| 4* | 4/19 | 0/0 | 0/0 | 0/0 | 0/0 | 0/0 (39) | - |
| 5 | 2/N/A | 4/N/A | 3/N/A | 3/N/A | 3/N/A | 3/22 (24) | - |
| 6 | 4/19 | 3/13 | 3/11.5 | N/A/N/A | N/A/N/A | 3/11.5 (12) | - |
| 7* | 4/24 | 1/9 | N/A/N/A | N/A/N/A | N/A/N/A | 1/9 (9) | - |
| 8 | 4/32 | 4/30.5 | N/A/N/A | N/A/N/A | N/A/N/A | 4/30.5 (6) | + |
| 9 | 4/13 | 4/14.5 | 4/14.5 | 4/14.5 | N/A/N/A | 4/14.5 (18) | - |
| 10 | 4/N/A | N/A/N/A | N/A/N/A | N/A/N/A | N/A/N/A | N/A/N/A | N/A |
| 11 | 2/N/A | N/A/N/A | N/A/N/A | N/A/N/A | N/A/N/A | N/A/N/A | N/A |
| 12 | 3/N/A | N/A/N/A | N/A/N/A | N/A/N/A | N/A/N/A | N/A/N/A | N/A |

**N/A, not available; mRS, modified Rankin Scale; SARA, Scale for the Assessment and Rating of Ataxia.** Patients marked with an asterisk (*) were previously reported [23–25].

Supplementary Table 3. Exploratory univariable analysis of clinical characteristics, cerebrospinal fluid findings, treatment variables, and outcomes according to prognosis in Japanese patients with anti-mGluR1 antibody-associated cerebellar ataxia.

| Variable | Good prognosis group (mRS ≤ 2 at last follow-up, n = 3) | Poor prognosis group (mRS ≥ 3 at last follow-up, n = 6) | *p* value | 95% CI |
| --- | --- | --- | --- | --- |
| Age (years) | 51 [51–58] | 64 [51–74] | 0.225 | [−22.5, 7.0] |
| Sex (female), n (%) | 2 (66.7%) | 3 (50.0%) | >0.999 | [0.11, 35.81] |
| Duration from onset to CSF sampling (months) | 1.0 [0.75–4.5] | 2.0 [0.25–12] | >0.999 | [−6.5, 4.25] |
| Cell counts (/μL) | 4 [1–6] | 4.5 [1–87] | 0.792 | [−43.0, 5.0] |
| Protein levels (mg/dL) | 26 [22–31] | 38.5 [29–337] | 0.070 | [−172.5, 0.0] |
| Oligoclonal band positive, n (%) | 1 (33.3%) | 5 (83.3%) | 0.226 | [0.004, 2.50] |
| Duration from onset to treatment (months) | 3.0 [1–6] | 2.5 [1–12] | 0.894 | [−4.5, 5.0] |
| Corticosteroid, n (%) | 1 (33.3%) | 6 (100%) | 0.083 | **–** |
| IVIG, n (%) | 3 (100%) | 4 (66.7%) | 0.500 | **–** |
| PP, n (%) | 0 | 3 (50.0%) | 0.464 | **–** |
| RTX, n (%) | 1 (33.3%) | 1 (16.7%) | >0.999 | [0.10, 62.61] |
| IS, n (%) | 1 (33.3%) | 2 (33.3%) | >0.999 | [0.05, 18.92] |
| mRS at presentation | 4 [2–4] | 4 [2–4] | 0.722 | [−2.0, 1.0] |
| SARA at presentation | 19 [8–24] | 18.5 [13–32], n = 5 | >0.999 | [−13.0, 9.0] |
| mRS at 6 months after onset | 1 [0–1] | 4 [3–4] | 0.021 | [−4.0, −2.0] |
| Relapse, n (%) | 0 | 2 (33.3%) | 0.500 | **–** |

**mRS, modified Rankin Scale; IVIG, intravenous immunoglobulin; IS, immunosuppressants; IVMP, intravenous methylprednisolone; PP, plasmapheresis; PSL, prednisolone; RTX, rituximab; SARA, Scale for the Assessment and Rating of Ataxia. Continuous variables are presented as median [range], and 95% CIs indicate the confidence intervals for the between-group differences. Categorical variables are presented as n (%), and 95% CIs indicate the confidence intervals for the odds ratios. Confidence intervals were not estimated when one cell contained zero.**
